# The Use of Cannabis for Headache Disorders

**DOI:** 10.1089/can.2016.0033

**Published:** 2017-04-01

**Authors:** Bryson C. Lochte, Alexander Beletsky, Nebiyou K. Samuel, Igor Grant

**Affiliations:** Department of Psychiatry, Center for Medicinal Cannabis Research, University of California, San Diego, La Jolla, California.

**Keywords:** cannabis, headache, therapy

## Abstract

Headache disorders are common, debilitating, and, in many cases, inadequately managed by existing treatments. Although clinical trials of cannabis for neuropathic pain have shown promising results, there has been limited research on its use, specifically for headache disorders. This review considers historical prescription practices, summarizes the existing reports on the use of cannabis for headache, and examines the preclinical literature exploring the role of exogenous and endogenous cannabinoids to alter headache pathophysiology. Currently, there is not enough evidence from well-designed clinical trials to support the use of cannabis for headache, but there are sufficient anecdotal and preliminary results, as well as plausible neurobiological mechanisms, to warrant properly designed clinical trials. Such trials are needed to determine short- and long-term efficacy for specific headache types, compatibility with existing treatments, optimal administration practices, as well as potential risks.

## Introduction

Headache is a major public health concern, with enormous individual and societal costs (estimated at $14.4 billion annually) due to decreased quality of life and disability.^1^ Each year, ∼47% of the population experience headache, including migraine (10%), tension-type headache (38%), and chronic daily headache (3%).^2^ A sexual dimorphism exists for headache disorders, with women 2–3 times more likely to experience migraine^3^ and 1.25 times more likely to experience tension-type headache than men.^4^

The present review will focus largely on migraine, tension-type headache, trigeminal autonomic cephalalgias (specifically cluster headache), and medication-overuse headache (MOH). Migraine is classified as a 4–72 h headache that is typically unilateral, pulsating, of moderate-to-severe intensity, and associated with photophobia and phonophobia.^5,6^ Tension-type headache is classified as frequent, infrequent, or chronic, typically presenting with bilateral tightening pain of mild-to-moderate intensity and lasting minutes to days.^6,7^ Cluster headache is defined as severe unilateral pain in orbital, temporal, and/or supraorbital locations, lasting 15–180 min and typically occurring frequently and at regular intervals.^6,8^ MOH is a chronic condition (occurs more than 15 days per month) that develops from frequent use of anti-headache medications.^6,9^

The pathophysiology of headache disorders is still under investigation. However, it is believed that migraine and cluster headaches are initiated in the brain in areas such as the hypothalamus, brainstem, or possibly cortex.^6^ Tension-type headaches can not only originate in the central nervous system but may also be triggered by myofascial tissue, often developing in response to stress.^10^ Regardless of origin, headaches usually involve overactivation of the trigeminovascular pathway, resulting in the release of vasoactive peptides, such as calcitonin gene-related peptide (CGRP) and substance P, as well as vasoactive mediators such as nitrous oxide (NO), which can lead to further sensitization of nociceptive receptors in the head and neck.^11^ Serotoninergic signaling, parasympathetic efferents, inflammation, and increased intracranial pressure also play important roles in headache disorders.^12,13^

Treatment depends on the underlying headache condition; however, some popular options include NSAIDs for mild headaches and triptans, anti-depressants, verapamil, or ergotamine for more severe or chronic headaches.^14^ These may be complemented by nonpharmacological interventions such as cognitive-behavioral therapy or relaxation training.^15^ Despite many treatment options, less than half of headache sufferers experience remission, and many continue to develop more severe or chronic headaches throughout their lifetime.^16^ Moreover, headache disorders are often underrecognized and undertreated.^17^ This current situation warrants an exploration of additional treatment options for headache disorders, with favorable side-effect profiles and efficacy in refractory patients.

One such option, cannabis, has been ignored in the United States for the past several decades but has an established history in the treatment of headaches. Assyrian manuscripts from the second millennium BCE recommended cannabis to “bind the temples,”^18^ and Ayurvedic preparations in the third and fourth centuries BCE were indicated for “diseases of the head” such as migraines.^19^ The prescription of cannabis was even recommended in ancient Greece, with Pedanius Dioscorides describing its use in his *De Maternia Medica* as a treatment for “pain of the ears.”^20^ Other citations documenting the use of cannabis for headache disorders arise from the ninth century in the Al-Aq-rabadhin Al-Saghir, the earliest known document of Arabic pharmacology.^19^ Further recommendations are found in Persian texts from the 10th^21^ and 17th centuries.^22^ Prominent physicians of the Middle Ages, including John Parkinson^23^ and Nicholas Culpeper,^24^ also recommended the use of cannabis for headache.

The reintroduction of cannabis to the West in 1839^25^ began a century of its use as an effective treatment for headache disorders^26^ until its illegalization in 1937.^27^ Notable physicians who espoused the benefits of cannabis for headache disorders included John Russell Reynolds, the personal physician of Queen Victoria,^28^ American neurologist Silas Weir Mitchell,^29^ the president of the New York Neurological Society Edouard C. Seguin,^19^ William Gowers, a founding father of modern neurology,^30^ and Sir William Osler, often considered the father of modern medicine.^31^

When cannabis was deemed illegal by the U.S. government, its therapeutic use and research into its medical potential was largely discontinued. To this day, there are few clinical investigations of the use of cannabis for headache; however, the studies that have emerged demonstrate potential efficacy. In addition, numerous pre-clinical investigations^18^ have validated the role of endocannabinoids in preventing headache pathophysiology, which suggests a mechanistic role of cannabis in the treatment of these disorders. Although the cannabis plant comprises more than 100 cannabinoids, there has been little study of the individual effects of these cannabinoids on headache disorders; therefore, the present review will focus largely on the clinical potential of the cannabis plant as a whole.

The present review has four unique aims: (1) Highlight common historical trends in the use of cannabis in the treatment of headache to inform future clinical guidelines. (2) Briefly present the current clinical literature on this topic, with a focus on more recent publications that have not been discussed in past reviews. (3) Compile various preclinical studies into a prospective integrated model outlining the role of cannabinoids in the modulation of headache pathogenesis. (4) Outline several^19,32–35^ future directions that warrant exploration based on the limited, but promising findings on this topic.

## Materials and Methods

The material presented was drawn from standard searches of the PubMed/National Library of Medicine database, influential sources of current medical literature, and past review articles. Search keywords included cannabis; cannabinoids; headache; migraine; cluster headache; medication-overuse headache; tetrahydrocannabinol; cannabidiol; clinical trial; placebo; and double blind. CliniacalTrials.gov was also queried for studies that have not yet been published. Individual articles were selected based on historical, clinical, or preclinical relevance to cannabinoids or cannabis as a treatment for headaches.

## Historical Use of Cannabis for Headache

Historical reports, though not ideal forms of evidence, are important resources for understanding the potential use of cannabis in the treatment of headache disorders. Clinical publications between 1839 and 1937 provide valuable insights into the most effective practices, challenges, and benefits during an era when cannabis was commonly used to treat headache. A summary of historical treatment practices using cannabis for migraines can be seen in Table 1. Historical sources indicate that cannabis was used as an effective prophylactic and abortive treatment for headache disorders. Although dosing varied among physicians, most prescribed alcohol extractions of the drug in the range of ¼ to ½ grain (16–32 mg).^28,32,36–40^ This dose was likely chosen to minimize the effects of intoxication while also providing effective therapeutic relief. Other providers suggested that doses should be progressively increased until modest effects of intoxication were felt.^19^ For prophylactic treatment, these doses were usually administered two to three times daily for weeks or even months.^28,32,36–38^ Acute treatment often involved higher doses taken as needed and, in some cases, smoked cannabis was recommended.^19,41–42^

**Table 1.** **Historical Reports of the Use of Cannabis as a Treatment for Headache (19th and Early 20th Century)**

Usage	Administration	Sample	Result	Source
Migraine	A: 0.03 fluid ounce of alcohol extract 1 h before pain onset	4 Case studies	Distinct termination of migraine. All patients experienced improvement, some were cured.	Donovan^41^
Migraine	A: 21.6 mg	2 Case studies	Immediate relief and elimination of headache for 14 months after treatment. No lasting harm.	Reynolds^27^
	P: 21.6 mg—three times daily			
Migraine/headache	P: 21.6 mg, 1–2 times per day (can increase to 43.2 mg)	9 Case studies and clinical experience	Responses in majority of cases. Usually lasting relief, sometimes curative. Palliative during headache.	Greene^35^; Russo^18^
Clavus hystericus and migraine	P: 21.6 mg to 43.2 mg every night	Textbook	Palliation even in severe cases.	Waring^36^
Migraine or sick headache	P: Taken before each meal (Women: 21.6 mg increased to 32.4 after 2–3 weeks; Men: 32.4 increased to 48.6)	Clinical experience	Majority of patients reported migraine relief for months.	Seguin (1878) cited in Russo^18^
Migraine or sick headache	A: 21.6–32.4 mg at beginning of attack.	Clinical experience	Found to be the most effective drug for migraine. Can abort attacks in some cases.	Ringer^37^
	P: 21.6–32.4 mg, 2–3 times daily, for weeks or months continuously.			
Migraine	P: 8.1–16.2 mg of solid extract twice a day.	Clinical experience	Helpful prophylactically and abortively, even in cases of migraine refractory to other treatments.	Hare^40^
	A: Take as needed			
Chronic daily headache	P: 21.6–32.4 mg (increasing if necessary), 2–3 times per day for weeks to months.	4 Case studies	Cured complaints in a majority of cases.	Mackenzie^38^
Migraine	P: 16.2 mg twice a day continuously	Short report	Given immediately will stop attack, given periodically will reduce severity and frequency.	Suckling^39^
	A: Take 16.2 mg during onset of attack			

A, abortive; P, prophylactic.

Early reports of cannabis for the treatment of headache appear to be largely positive, with many patients experiencing a decrease in the frequency and intensity of their headache episodes. In some cases, headache was cured entirely even after cannabis discontinuation.^28,32,36–42^ Furthermore, these early clinical reports praise the apparent safety of long-term cannabis use, as well as its added benefits of mollifying the nausea and anxiety that often accompany headaches. A common emphasis was placed on the importance of specific purity, preservation, and administration of the cannabis as well as patient adherence in the efficacy of treatment.

## Clinical Studies on Cannabis Use for Headache

The schedule 1 classification of marijuana in 1970 has made rigorous clinical studies on the treatment efficacy of this substance difficult. Currently, there are no placebo-controlled clinical studies examining the use of cannabis for headache; nevertheless, there have been a number of other studies published that give insight into its therapeutic efficacy (Table 2).^19,43–58^ However, care should be taken when interpreting the findings from these studies. With one exception,^53^ these studies did not include a control group, and given that the placebo effect can be altered by the context of treatment,^59^ it is reasonable to expect a significant placebo response given the pre-existing public popularity and notoriety of cannabis. Moreover, self-reports and case studies may have a bias toward immediate improvement without awareness of possible dependence, rebound, or withdrawal responses, which are important concerns in headache treatment.^60^ In fact, studies show that headache can be induced in 23.2% patients undergoing cannabis withdrawal.^61^

**Table 2.** **Clinical Reports of the Use of Cannabis or Exogenous Cannabinoids as a Treatment for Headache**

Subject population	Type of study	Significant findings	Source
3 Chronic smokers	Case series	Migraines after cannabis cessation. Remission of headache with return to use in one patient.	El-Mallakh^42^
Patient with migraine	Case report	Women found superior relief of migraine with cannabis compared with beta-blockers, opiates, and ergots.	Petro (1997) cited in Russo^18^
Patient with migraine	Case report	18 years of treatment failure with standard pharmaceuticals, found success with smoked cannabis.	Grinspoon and Bakalar^45^
Patient with migraine	Case report	Successful treatment with cannabis that did not produce inebriation.	Terwur (1997) cited in Russo^18^
121 Patients prescribed cannabis for migraine	Retrospective study	Migraine occurrences decreased from 10.4 to 4.6 per month; 39.7% had a positive effect, 19.8% had decreased frequency, and 11.6% had aborted pain.	Rhyne et al.^46^
5 Cases of chronic migraine headache	Case series	All cases successfully treated with dronabinol or cannabis. In one case, cannabis improved response more than dronabinol. In three cases, cannabis was used to abort headache in the prodromal phase.	Mikuriya^48^
1655 Patients seeking physician recommendation for medical cannabis	Survey	40.8% of applicants reported improvement of headache symptoms with cannabis.	Nunberg et al.^49^
3 Subjects with chronic headaches	Case series	Smoking cannabis caused relief similar or greater than ergotamine and aspirin.	Noyes Jr. and Baram^50^
30 Outpatients with medication-overuse headache	Clinical Trial (RDAC—Crossover)	Nabilone was superior to ibuprofen in reducing pain intensity, analgesic intake, and medication dependence while improving quality of life.	Pini et al.^52^
Patient with refractory cluster headache	Case report	Smoked cannabis or dronabinol at the beginning of cluster headache provided complete immediate headache relief.	Robbins et al.^53^
113 Patients with chronic cluster headache	Survey	26% regularly used cannabis. Use as treatment unknown.	Donnet et al.^54^
139 Patients with chronic cluster headache	Survey	Overall, 45.3% had used cannabis, and 19.4% had used it to treat cluster headache; 25.9% found efficacy, and the remainder found variable or negative effects.	Leroux et al.^55^
Patient with pseudotumor cerebri	Case report	Complete resolution of headache with smoking cannabis in <5 min without reoccurrence.	Evans and Ramadan^56^
112 Patients with MS-associated trigeminal neuralgia	Survey	Overall, 70% found relief from trigeminal neuralgia, and 90% found chronic pain relief.	Consroe et al.^57^

MS, multiple sclerosis.

Nabilone, a synthetic cannabinoid mimicking tetrahydrocannabinol (THC), has been shown to decrease analgesic intake while reducing MOH pain in a double-blind, placebo-controlled trial.^53^ In this study, 26 patients with treatment refractory MOH completed a course of either nabilone (0.5 mg) or ibuprofen (400 mg) for 8 weeks, then after a week-long washout period, completed a second 8-week course of the previously excluded medication. Oral cannabinoid administration was chosen over an oromusocal THC spray, both because oral administration avoids the concentration peaks that can lead to euphoric effects and because chronic administration better overcomes individual differences in bioavailability. Although both substances showed improvement from baseline, nabilone was significantly more effective than ibuprofen in reducing pain intensity, analgesic intake, and medication dependence, as well as in improving quality of life. This study also examined the safety of nabilone as a treatment for headache and found that patients only experienced mild adverse effects that disappeared after discontinuation of the medication. The results of this study are significant, especially given that MOH is exacerbated by many pharmacological treatments. This study also highlights the potential value of cannabis in combination therapies, as a supplement to traditional treatments, or as a secondary treatment in refractory cases. Currently, a multicenter, double-blind, placebo-controlled study is being performed to examine the safety and efficacy of a dronabinol, or synthetic THC, metered dose inhaler for the treatment of migraine (clincaltrials.gov, NCT Identifier: NCT00123201). When published, this study could give valuable insights into the efficacy and risks of cannabinoids for the treatment of migraines.

Cannabis and cannabinoids have been studied clinically for other conditions, showing efficacy in the treatment of neuropathic/chronic pain, spasticity, and nausea.^62–66^ These three conditions are associated mechanistically and qualitatively with the experience of headache and, although the clinical literature for each of these conditions exceeds the scope of this review, it is plausible that their efficacy will carry over in the treatment of headache disorders as well. For example, the analgesic properties of cannabis seen in the treatment of neuropathic pain will likely apply to chronic headache, the antispasmodic properties seen in the treatment of multiple sclerosis could apply to muscle strain known to induce tension headaches, and the antiemetic properties seen in the treatment of chemotherapy-associated nausea might also palliate migraine-induced nausea.

Many individuals are currently using cannabis for the treatment of migraine and headache with positive results. In a survey of nine California clinics (*N*=1746), physicians recorded headaches and migraines as a reason for approving a medical marijuana ID card in 2.7% of cases, and 40.7% patients self-reported that cannabis had therapeutic benefits for headaches and migraines. In another California survey of 7525 patients, 8.43% of patients reported that they were using medical cannabis to treat migraines. Another survey of 1430 patients found that 9% of patients were using medical cannabis to treat migraines (subdivided into 7.5% for classical migraines, 1% for cluster headaches, and 0.5% for others). Other studies have reported the use of cannabis for migraine or headache relief, with specific estimates including 5% (*N*=24,800) and 6.6% (*N*=128) for migraines and 3.6% (*N*=128) and 7.4% (*N*=217) for headache.

Other studies have looked specifically at the change in the occurrence of headache disorders with use of cannabis.^52^ One retrospective study described 121 patients who received cannabis for migraine treatment, among whom 85.1% of these patients reported a reduction in migraine frequency.^47^ The mean number of migraines at the initial visit was 10.4, falling to 4.6 at follow-up visits after cannabis treatment. Moreover, 11.6% of the patients found that, when smoked, cannabis could effectively arrest the generation of a migraine. These results indicate that cannabis may be an effective treatment option for certain migraine sufferers.

Reports from 139 cluster headache patients^56^ indicate that cannabis could have value in treating a portion (25.9%) of these patients as well. However, cannabis was reported to provoke cluster headache attacks in some patients (22.4%) as well. One possible explanation for this provoking effect is that cannabis is known to increase heart rate, increase blood pressure, and cause systemic vasodilation.^67^ Cluster headache sufferers seem to be highly sensitive to vasodilation of the carotid tree and increased oxygen demands, findings that are supported by evidence that alcohol is a reliable trigger and supplemental oxygen is an effective abortive therapy.^68^ The increased oxygen demand and/or the vasodilation effects of cannabis could theoretically be responsible for this exacerbation in some cluster headache sufferers. Interestingly, cluster headaches appear to show improvement with treatment using hallucinogens such as d-lysergic acid amide (ergine or LSA), psilocybin, and lysergic acid diethylamide (LSD).^33^ As such, it is possible that the psychoactive properties of THC could play a role in the treatment of cluster headaches.

Case reports also give insights into the mechanisms behind the anti-headache action of cannabis. Smoking cannabis has been reported to relieve pain associated with pseudotumor cerebri,^57^ a condition that is characterized by an increase in the intracranial pressure of an uncertain etiology. This suggests that the therapeutic effect of cannabis in some headache conditions could be a result of reducing intracranial pressure. In fact, dexanabinol, a synthetic cannabinoid, has been found to relieve intracranial pressure and improve outcomes after traumatic brain injury.^69^

## Cannabinoids and Headache Pathophysiology

**FIG. 1.**
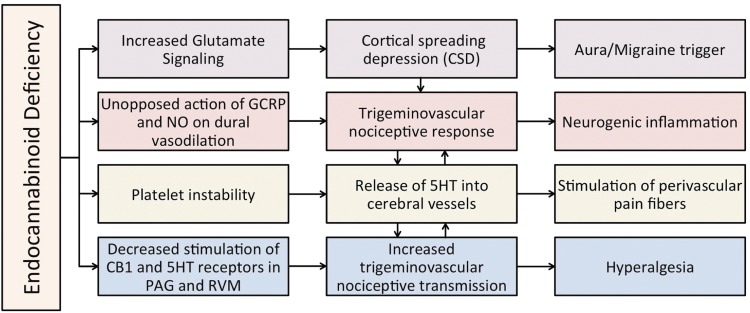
Proposed model of the influence of cannabinoids on headache pathogenesis. Each branch corresponds to a mechanistic category listed in Table 3. Orange=systemic; purple=cortex; red=vasculature; green=platelets; blue=brainstem.

The pathophysiological mechanisms of many headache disorders are not entirely understood. Nevertheless, preclinical data examining the effects of endocannabinoids on the neurological and vascular systems demonstrate the influence of endocannabinoids in modulating several major components of migraine pathogenesis (Table 3 and Fig. 1).^35,70–85^

**Table 3.** **Studies on the Role of Cannabinoids in Headache Pathogenesis**

Mechanistic category	Significant findings	Source
Systemic	Variants in the *cnr1* gene (encodes for the CB1 receptor) resulting in decreased expression of CB1 associated with migraine and trigeminovascular activation.	Juhasz et al.^75^
	Levels of AEA are decreased in the cerebrospinal fluid of individuals with chronic migraine, whereas levels of CGRP and NO (inhibited by AEA) are increased.	Sarchielli et al.^76^
	Endocannabinoid deficiency theorized as a possible cause for migraine and other chronic pain disorders, including chronic migraine and medication-overuse headache.	Cupini et al.^77^
	Female migraineurs have increased FAAH and EMT activities.	Cupini et al.^78^
Cortex	CB1 agonists suppress glutamatergic neurotransmission by inhibiting NMDA receptors.	Hampson et al.^79^
	CB1 agonists suppress CSD.	Kazemi et al.^80^
Vasculature	AEA reduced nitroglycerin-induced neuronal activation in the nucleus trigeminalis caudalis.	Greco et al.^81^
	AEA inhibits dural blood vessel dilation induced by CGRP, capsaicin, and NO (model of trigeminovascular nociceptive response). AEA also prevented the release of NO by CGRP in dural arteries.	Akerman et al.^82^
	Hyperalgesia induced by NO nearly eliminated in FAAH deletion or with FAAH inhibitor.	Nozaki et al.^83^
	AEA activates TRPV1 on afferent trigeminal ganglion neurons, leading to CGRP release and cranial vasodilation.	Akerman et al.^93^
	CBD is TRPV1 agonist. Could desensitize receptor and inhibit pathophysiological mechanism of headache.	Bisogno et al.^84^
Platelets	Endocannabinoid levels reduced in platelets of patients with migraine.	Rossi et al.^85^
	Platelets of women with migraine showed increased activity of FAAH when compared with men with migraine.	Cupini et al.^78^
	Cannabinoid compounds may stabilize and inhibit 5HT release from platelets during a migraine.	Volfe et al.^96^
Brainstem	CB1 receptor activation in PAG and RVM leads to top-down modulation of pain.	Kelly and Chapman^86^
	AEA potentiates 5HT1A and inhibits 5HT2A receptors.	Boger et al.^87^
	Endocannabinoids interact with serotonergic neurons in the brainstem dorsal raphe to modulate pain mechanisms.	Haj-Dahmane and Shen^88^
	NO increases activity of FAAH, leading to increased breakdown of endocannabinoids in the midbrain/PAG.	Greco et al.^30^
	Elevation of endocannabinoid levels in the PAG modulates descending nociceptive pathways via CB1 and TRPV1.	Maione et al.^89^
	CB1 receptor activation in the vlPAG attenuated trigeminocervical complex activity. This effect was inhibited by the addition of the CB1 receptor antagonist or the 5HT1B/1D receptor antagonist.	Akerman et al.^90^

AEA, anandamide; CB1, cannabinoid receptor type 1; CBD, cannabidiol; CGRP, calcitonin gene-related peptide; CSD, cortical spreading depression; EMT, endocannabinoid membrane transporter; FAAH, fatty acid amide hydrolase; NMDA, *N*-methyl-d-aspartate; NO, nitrous oxide; PAG, periaqueductal gray; RVM, rostral ventromedial medulla; vlPAG, ventrolateral PAG.

### Underlying cause of headaches

Various genetic factors can predispose individuals to migraines. For example, studies have shown that a decrease in expression of the *cnr1* gene, which encodes the cannabinoid receptor type 1 (CB1) receptor, is associated with migraine and trigeminovascular activation.^70^ Women who experience migraine also have increased activities of fatty acid amide hydrolase (FAAH), an enzyme used to degrade the endocannabinoid anandamide (AEA), and the endocannabinoid membrane transporter (EMT), a membrane transporter for AEA, leading to an overall decrease in levels of endocannabinoids.^73^ This finding could partially explain the increased prevalence of migraines in women. An examination of cerebrospinal fluid shows that individuals who experience migraines have decreased levels of AEA and increased levels of CGRP and NO (normally inhibited by AEA). These findings support the proposed theory that alterations in endocannabinoid function with reductions in endocannabinoids such as AEA may be one of the mechanisms underlying migraine. A feature of headache disorders is that they are highly associated with other comorbidities, including anxiety and mood disorders, allergies, chronic pain disorders, and epilepsy.^86^ The endocannabinoid deficiency hypothesis provides a possible mechanism underlying not only migraine but also diseases such as fibromyalgia and irritable bowel syndrome.^72^ Although the endocannabinoid deficiency hypothesis is still speculative and in need of further study, it suggests that exogenous stimulators of the endocannabinoid system, such as cannabis, could treat these diseases at their source.^87^

### Glutamate signaling

One of the first subjective indicators of a migraine is the occurrence of an aura, a perceptual abnormality that often precedes a migraine attack. A wave of electrophysiological hyperactivity followed by inhibition, known as cortical spreading depression (CSD), is considered the neurobiological event underlying the migraine aura. CSD has been shown to be a result of excessive glutamate signaling, and one effect of endocannabinoids is the suppression of glutamate signaling via the inhibition of NMDA receptors.^74^ In fact, suppression of CSD has been achieved by THC and cannabinoid CB1 agonist activation of CB1 receptors in murine models.^75^ This suggests a use for cannabis in the prevention of the initial mechanisms triggering a migraine aura and the subsequent pain.

### Trigeminovascular activation

Another component of most headache disorders is overactivation of the trigeminovascular system, the primary sensory nerve tree for the head. One of the most reliable triggers for migraine is NO. Studies have demonstrated the role of endocannabinoids in inhibiting NO.^76^ Moreover, AEA has been shown to inhibit dural vascular dilation caused by NO, CGRP, capsaicin, and electric stimulation.^77^ This effect may seem paradoxical, as cannabis is a known vasodilator and AEA acts through the vanilloid receptor TRPV1 to cause dilation of the cranial blood vessels.^88^ However, because cannabinoids such as THC bind preferentially with CB1 receptors over TRPV1,^89^ concentration could determine whether cannabinoids have a vasodilatory or vasoconstrictive effect. For example, at low concentrations, AEA inhibits neurogenic vasodilation, but at higher concentrations, AEA will begin binding with TRPV1 and induce vasodilation.^88^ This concentration-dependent activation of TRPV1 may underlie some of the paradoxical (e.g., anxiogenic, hyperalgesic) effects of THC seen at higher doses. Moreover, vasodilation is not necessarily pathogenic for headaches, and endocannabinoid-induced vasodilation could desensitize the vasculature to known headache progenitors, such as NO. Interestingly, NO appears to also exert nociceptive effects through FAAH, as deletion of an FAAH inhibitor or addition of an FAAH inhibitor prevents nociceptive reaction to NO.^78^ One could postulate a possible feedback effect wherein NO and FAAH overpower endocannabinoids to illicit pain.

### Platelet stabilization

The hematological properties within the dilated cranial blood vessels themselves may also play an important role in the pathophysiology of migraine. Endocannabinoid levels are reduced in the platelets of migraine patients,^80^ and women with migraine show increased FAAH and EMT activation in their platelets.^73^ Research has indicated that migraine might, in part, result from serotonin that is released from aggregating platelets,^90^ a theory that is supported by the efficacy of antiplatelet medications in some migraine sufferers. Cannabinoid compounds have been shown to stabilize platelets and prevent release of serotonin from platelets during a migraine.^91^

### Modulation of afferent nociceptive signals

Endocannabinoids have a well-established role in the modulation of pain signals at the spinal level^81^ and contribute to the descending modulation of pain through brainstem nuclei.^92^ Endocannabinoids also inhibit trigeminovascular nociceptive processing with dural inputs.^93^ The activation of the trigeminovascular system leads to activation of cutaneous evoked afferent A and C-fibers.^93^ Endocannabinoids inhibit these signals via projections from the periaqueductal gray (PAG) and rostral ventral medulla.^94–96^ CB1 receptor activation in the ventrolateral PAG has also been shown to modulate nociceptive trigeminovascular transmission in the trigeminocervical complex via activation of 5HT1B/1D receptors.^85^ Endocannabinoids also influence serotonergic neurons within the brainstem dorsal raphe to modulate pain.^83^

Triptans, one of the most effective abortive treatments for migraine and cluster headaches, are believed to act through agonist effects on 5HT1B/1D receptors on the nerve endings in cranial blood vessels,^97^ as well as brainstem regions, including the PAG,^85^ resulting in decreased release of pro-inflammatory neuropeptides such as substance P and CGRP and attenuation of dural nociceptive responses. Since 5HT1B/1D antagonists can inhibit the CB1 modulation of nociceptive trigeminovascualr signals, triptans may induce their anti-migraine effects by activating endocannabinoid-containing neurons in the PAG.^85^

## Discussion

Headache disorders are common, painful, and disabling; moreover, treatment for these disorders is inadequate for many sufferers. Before cannabis was made illegal, many prominent physicians praised its use in the treatment of headache disorders. Reports from this period emphasize the administration of consistent and uniform doses and the titration of doses to minimize intoxication. For prophylactic treatment, cannabis was typically given orally two to three times per day, for weeks or even months,^28,32,36–38^ and for abortive treatment, cannabis was given at higher oral doses or smoked.^19,41,42^ If cannabis is to be reconsidered as a treatment for headache, considering this historical perspective could improve the efficacy of treatments and help inform future research.

Although there have not been any clinical trials of cannabis as a treatment for headache to date, reports indicate that cannabis is commonly used by patients to self-medicate for headache disorders. A retrospective analysis has shown a significant impact of cannabis in treating migraine^47^ and a clinical trial of a synthetic cannabinoid showed efficacy for MOH,^53^ but properly designed placebo-controlled trials are needed to determine the true efficacy and complications of cannabis treatment for headache disorders.

Preclinical studies examining the role of the endocannabinoid system in migraine pathogenesis also suggest a potential therapeutic value for cannabis in the treatment of headache. It has been postulated that a general deficiency in endocannabinoid tone could underlie headache disorders.^72^ Cannabis also shows potential to interrupt specific stages in the pathogenesis of headaches, including glutamate signaling leading to CSD,^75^ cranial blood vessel dilation caused by NO and CGRP,^77^ serotonin release from platelets,^91^ and afferent trigeminovascular nociceptive inputs.^85^ Although these studies have suggested an interesting relationship between endocannabinoids and some pathogenic processes of headache disorders, the mechanistic role of cannabis in preventing headache disorders remains speculative.

The studies presented in this review indicate the importance of further well-designed clinical trials of the efficacy of cannabis in the treatment of headache disorders. Because there are still many obstacles present in constructing double-blind placebo-controlled clinical trials of cannabis, the following list outlines various other potential future investigations and recommendations based on the findings presented in this review.

1. The development of dosing and treatment guidelines for the use of cannabis in the treatment of headache disorders. Physicians should consider discussing dosing strategies when recommending cannabis as headache treatment, with the aim of maximizing efficacy and minimizing harm. A focus on dose consistency through the use of oral cannabinoids or metered-dose inhalers could benefit future clinical trials by allowing for easier blinding and placebo control. Moreover, the use of oral cannabinoids could have unique benefits in the prophylactic treatment of headache, because it could avoid concentration peaks and individual differences in bioavailability.
2. Investigation of the anti-headache effect of cannabidiol (CBD). This review found no available information on the use of CBD as a treatment for headache. Nevertheless, CBD has shown efficacy for headache-related conditions (i.e., anxiety),^98^ has demonstrated an analgesic role associated with TRPV1 receptors,^99^ and can serve as a 5HT1a receptor agonist.^100^
3. Identification of variables that could predict treatment receptivity in headache patients. This could include stratification of headache disorders or patients based on sex, genetics, metabolic function, or neuronal biomarkers.
4. Investigation of the long-term risks of cannabis treatment for headaches. This should aim at quantifying any side effects, withdrawal symptoms, dependence, refractory headaches, or negative outcomes from cannabis treatment for headaches.
5. Evaluation of other anti-headache drugs that target the endocannabinoid system. Preclinical data suggest the possible use of FAAH or EMT inhibitors, which might have unique efficacy in female migraineurs.
6. Evaluation of cannabis in combination treatment (with analgesic or other anti-headache medications) or as a second-line treatment in patients who are refractory to traditional medications.

## Conclusion

The present review examines the historical guidelines for cannabis treatment of headache, available clinical data on the use of cannabis for headache, and preclinical literature on the role of the endocannabinoid system in headache pathophysiology. From this examination, various methodological recommendations are made for future studies and potentially novel treatment practices are considered. Although placebo-controlled clinical trials are still needed to appropriately determine efficacy, it appears likely that cannabis will emerge as a potential treatment for some headache sufferers.

## Abbreviations Used

5HT: serotonin
5HT1A: 5-hydroxytryptamine receptor 1A
5HT1B/1D: 5-hydroxytryptamine receptor 1B/1D
5HT2A: 5-hydroxytryptamine receptor 2A
A: abortive
AEA: anandamide
BCE: Before Common Era
CB1: cannabinoid receptor type 1
CBD: cannabidiol
CGRP: calcitonin gene-related peptide
CSD: cortical spreading depression
EMT: endocannabinoid membrane transporter
FAAH: fatty acid amide hydrolase
MOH: medication-overuse headache
MS: multiple sclerosis
NMDA: *N*-methyl-d-aspartate
NO: nitrous oxide
NSAIDs: nonsteroidal anti-inflammatory drugs
P: prophylactic
PAG: periaqueductal gray
RVM: rostral ventromedial medulla
THC: tetrahydrocannabinol
TRPV1: transient receptor potential cation channel subfamily V member 1
vlPAG: ventrolateral PAG
